# Amphetamine Stimulates Endocytosis of the Norepinephrine and Neuronal Glutamate Transporters in Cultured Locus Coeruleus Neurons

**DOI:** 10.1007/s11064-019-02939-6

**Published:** 2020-01-07

**Authors:** Suzanne M. Underhill, Mark S. Colt, Susan G. Amara

**Affiliations:** 1 Laboratory of Molecular and Cellular Neurobiology, National Institute of Mental Health, National Institutes of Health, 35 Convent Drive, Room 3A:209, Bethesda, MD 20892 USA; 2 Present Address: University of Southern California Graduate Program, Los Angeles, CA 90033 USA

**Keywords:** Norepinephrine, TAAR1, RhoA, Locus coeruleus, EAAT3, NET

## Abstract

Amphetamines and amphetamine-derivatives elevate neurotransmitter concentrations by competing with endogenous biogenic amines for reuptake. In addition, AMPHs have been shown to activate endocytosis of the dopamine transporter (DAT) which further elevates extracellular dopamine (DA). We previously found that the biochemical cascade leading to this cellular process involves entry of AMPH into the cell through the DAT, stimulation of an intracellular trace amine-associated receptor, TAAR1, and activation of the small GTPase, RhoA. We also showed that the neuronal glutamate transporter, EAAT3, undergoes endocytosis via the same cascade in DA neurons, leading to potentiation of glutamatergic inputs. Since AMPH is a transported inhibitor of both DAT and the norepinephrine transporter (NET), and EAAT3 is also expressed in norepinephrine (NE) neurons, we explored the possibility that this signaling cascade occurs in NE neurons. We found that AMPH can cause endocytosis of NET as well as EAAT3 in NE neurons. NET endocytosis is dependent on TAAR1, RhoA, intracellular calcium and CaMKII activation, similar to DAT. However, EAAT3 endocytosis is similar in all regards except its dependence upon CaMKII activation. RhoA activation is dependent on calcium, but not CaMKII, explaining a divergence in AMPH-mediated endocytosis of DAT and NET from that of EAAT3. These data indicate that AMPHs and other TAAR1 agonists can affect glutamate signaling through internalization of EAAT3 in NE as well as DA neurons.

## Introduction

The biogenic amine neurotransmitters including dopamine (DA), norepinephrine (NE) and serotonin (5-HT) are well-known to play important roles in regulation of mood and in neuropsychiatric disorders including addiction, depression, ADHD and schizophrenia. Recent work has centered on the modulation of DA and 5-HT signaling to treat these conditions, however, the NE system, which contributes critically to the modulation of mood and attention, also remains an important target for many psychostimulant and antidepressant medications. A number of drugs, including amphetamine and several amphetamine-derivatives, are potent regulators of both NE and DA systems, but whether these drugs alter neurotransmission through similar mechanisms in the two systems has not been fully explored.

Recent work from our lab has focused on how amphetamines (AMPHs) regulate internalization of the DA transporter (DAT) and a neuronal glutamate transporter (EAAT3) in DA neurons [1]. AMPHs are used clinically for the treatment of ADHD, obesity and narcolepsy, but many details surrounding the molecular, cellular and neurophysiological mechanisms that underlie their actions remain elusive. In our studies in DA neurons, we observed a cascade of signaling events that occur in response to AMPH, which ensue once the psychostimulant enters the cytoplasm through the dopamine transporter, DAT [1]. In previous studies, we and others observed that the route of AMPH entry into the cell did not matter—it could enter through either DAT or NET [1] or by direct injection into cells [2]. Once inside the cells, AMPHs bind to an intracellular receptor, the Trace Amine Associated Receptor 1 (TAAR1) that initiates the biochemical cascades [3]. Once activated by AMPH, a subset of intracellular TAAR1 receptors couples to the activation of the small GTPase RhoA, activating a pathway that leads to the internalization of the DAT, as well as of EAAT3 [4]. Interestingly, AMPH also activates another population of TAAR1 receptors that couple through an adenylyl cyclase/cAMP/PKA pathway, which subsequently inhibits RhoA through a negative feedback loop. This second set of events leads to the inactivation of RhoA by direct PKA-mediated phosphorylation, which in turn limits the RhoA-dependent internalization of DAT and EAAT3. DAT internalization contributes to an increase in extracellular dopamine in response to AMPH that is generally attributed the effects of psychostimulants. In parallel, EAAT3 internalization activated by AMPH and closely-related compounds leads to an increase in extracellular glutamate and enhanced glutamatergic neurotransmission that also contributes to the behavioral outcomes of these drugs.

Most of this previous work focused on DAT in DA neurons, but we were interested in understanding whether AMPHs might have similar effects on intracellular signaling pathways and transporter trafficking in NE neurons. Upon the cloning of NET, it was demonstrated that NET actually has a higher apparent affinity (K_T_) for NE, DA and AMPH, but a low transport capacity (V_max_) than DAT [5]. Despite this, AMPH has potent actions to block reuptake by NET and has been shown to stimulate internalization of NET in recombinant systems [6], though the precise mechanism in NE neurons is yet unclear. We were further curious about the effects of EAAT3 and glutamatergic neurotransmission in NE neurons which also express EAAT3 [7] and may be subject to the same regulation that we observed in DA neurons. In this study, we investigated the actions of AMPH in NE neurons derived from the locus coeruleus (LC) by examining its ability to stimulate TAAR1 receptors, to activate RhoA and PKA pathways and to trigger internalization of NET and EAAT3.

## Results

### DAT and NET Transport Both NE and DA

Many studies have established the apparent affinity (K_T_) and maximum transport velocity (V_max_) of DAT and NET for various amine substrates [5, 8] including their two respective neurotransmitters, and we wanted to establish direct comparisons of DAT and NET for DA, NE and AMPH in our transiently transfected cell culture model. Therefore, HEK293 cells were transfected with GFP-tagged DAT or NET and the transport of either ^3^H-DA, NE or AMPH was measured over 10 min. Data were normalized to the amount of transporter that was localized to the cell surface, as assessed by biotinylation assays for GFP. The transfection efficiency of GFP-NET was 93.24% that of GFP-DAT, that was not statistically different (p = 0.476 by paired *t* test). The V_max_’s for transport of DA or of NE by the DAT were considerably higher than those for the NET (Table 1). However, NET had a higher affinity (lower K_T_) for DA and NE than DAT, similar to previous observations. The maximal velocities for AMPH transport were not dramatically different between the two carriers, though the NET has a higher apparent affinity for AMPH.

**Table 1:** Kinetics of monoamine transport by DAT and NET

	DAT			NET		
	DA	NE	AMPH	DA	NE	AMPH
V_max_ (SEM)	1.335 (0.056)	1.536 (0.071)	1.679 (0.150)	0.290 (0.026)	0.598 (0.039)	1.28 (0.118)
K_T_ (SEM)	3.423 (0.341)	5.220 (0.497)	1.435 (0.406)	1.389 (0.379)	1.144 (0.248)	0.897 (0.292)

Kinetics determined by Michaelis–Menten saturation curves

V_max_ data presented in arbitrary units (AU) defined after normalization for expression

K_T_ data is in µM

### Like the DAT, NET also Internalizes in Response to AMPH

Despite the homologies between these two Slc6 family members, the differences in substrate affinities, transport capacities and pharmacological sensitivities of the two catecholamine neurotransporters suggest that there may also be important differences in their roles in the brain. DAT and NET both transport AMPH, although NET has an approximate 1.5-fold higher apparent affinity (Table 1). Both transporters are also reported to be internalized in response to pretreatment with AMPH [2, 6], but it whether they are regulated through the same mechanism remains unclear.

**Fig. 1:**
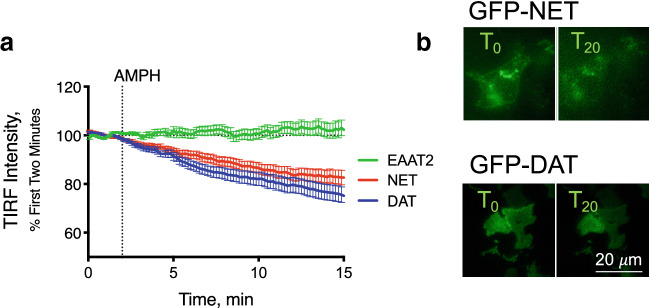
Internalization of DAT and NET in response to AMPH Membrane expression of GFP-tagged NET (red), DAT (blue) or the glutamate transporter, EAAT2 (green), were measured by total internal reflection fluorescence (TIRF) microscopy. After a 2-min baseline, AMPH (10 μM) was applied. Both NET and DAT internalized in response to AMPH while EAAT2 was unaffected (**a**). Representative images of HEK293 cells expressing GFP-tagged NET or DAT before and 20 min after AMPH application (**b**). Scale bar is 20 μm for all images (Color figure online)

We examined HEK293 cells expressing either GFP-tagged NET or DAT using total internal reflection fluorescence (TIRF) microscopy and measured the membrane localization of the carriers after treatment with (+)-amphetamine hemisulfate, 10 μM (Fig. 1). The intensity profiles (Fig. 1a) and time-lapse images (Fig. 1b) indicate that NET and DAT are both internalized in response to treatment with AMPH. In other experiments, the glial glutamate transporter, EAAT2, maintained its presence at the cell membrane indicating that in this model, AMPH-stimulated trafficking is specific for the catecholamine transporters (data not shown).

### AMPH-mediated NET Internalization in Primary Culture of NE Neurons

**Fig. 2:**
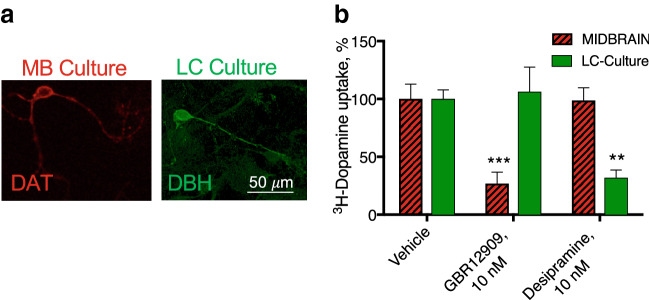
DAT and NET-mediated uptake in midbrain (MB) and locus coeruleus (LC) cultures. **a** DA neurons in the midbrain culture express DAT (red) and NE cultures in the LC cultures expressed dopamine –β hydroxylase (DBH, green). **b**^3^H-DA uptake was measured in LC and MB cultures. DA uptake in the midbrain cultures was sensitive to the DAT inhibitor, GBR12909, indicating predominantly DAT-mediated activity in those cultures. LC cultures, however, were unaffected by GBR12909 and instead sensitive to the NET-selective inhibitor desipramine (**p < 0.01 and ***p < 0.001 by one-way ANOVA compared to vehicle control) (Color figure online)

HEK293 cells are relatively easy to transfect with fluorophore-tagged carriers and amenable to many types of assays, however they may not reflect the physiological responses seen in neurons. We had previously made use of primary midbrain cultures to examine DAT function [1]. Midbrain cultures were derived from E15 mice and had ^3^H-DA uptake that was sensitive to GBR12909 at doses that were specific to DAT, indicating DAT-mediated uptake. In order to examine the effects of AMPH on NE neurons, we developed cultures from the brain tissue more posterior to the midbrain, containing the developing locus coeruleus (LC). After 2–5 weeks in culture, 18 ± 3% of the neurons in these cultures were positive for the NE enzyme dopamine β-hydroxylase (DBE, Fig. 2a). These cultures were also capable of ^3^H-DA uptake, but this activity, in contrast to the ^3^H-DA uptake in midbrain cultures, was not blocked by the GBR12909 and instead was blocked by the NET-specific inhibitor desipramine indicating NET-mediated transporter activity in these cultures (Fig. 2b). These data indicate that we developed a NE primary cell culture in which we could observe the activity of NET in endogenous cells.

### TAAR1-stimulated RhoA Activation Mediates AMPH-stimulated Internalization of Both Catecholamine Transporters

**Fig. 3:**
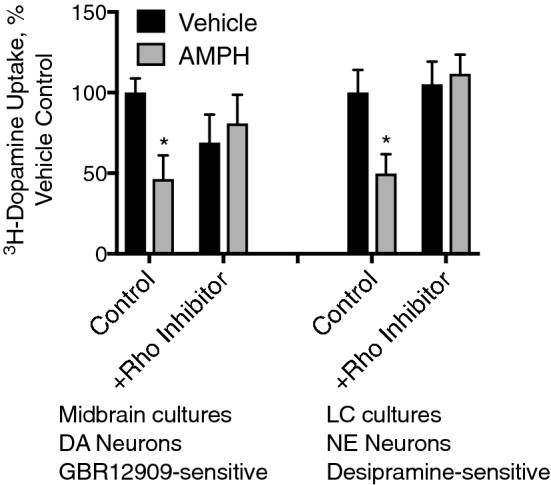
AMPH-mediated DAT and NET internalization in primary cultures is mediated by the small GTPase RhoA In MB cultures, DAT-mediated uptake (with the GBR12909 defined background already subtracted), a 30-min pre-treatment with AMPH (10 μM), leads to a dramatic loss of DAT transport capacity. However, this sensitivity to AMPH is abolished by pre-application of the RhoA Inhibitor I indicating a process mediated by the small GTPase RhoA. Similarly, NET-mediated, desipramine-sensitive ^3^H-DA transport in LC cultures was also sensitive to the AMPH pretreatment and similarly this loss of function was abolished by the RhoA inhibitor (*p < 0.05 by two-way ANOVA compared to vehicle control. F_(3, 33)_ = 3.295)

Using these primary culture models, we re-examined the mechanism of action of AMPH. Similar to our previous observations, the DAT-mediated, GBR12909-sensitive DA uptake was decreased in response to pre-treatment of the cultures with AMPH, 10 μM for 30 min. Previously, this loss of activity had been determined to be due to a RhoA GTPase-dependent internalization which could be blocked by dominant-negative RhoA constructs and by genetic introduction of the exotoxin C3, a clostridial exotoxin that inactivates RhoA by ADP-ribosylation [1]. Here, we used a recently developed RhoA inhibitor (CT04, Cytoskeleton, Inc.) also using C3, but attached to a cell-permeable moiety, and showed that it could block the effects of the AMPH pretreatment in DAT trafficking in DA neurons (Fig. 3). Similarly, in the LC cultures, the desipramine-sensitive ^3^H-DA uptake was decreased in response to AMPH pretreatment, indicating that, like the DAT in MB cultures, the NET in LC neurons is endocytosed in response to AMPH treatment. These effects of AMPH on the NET in NE neurons was also blocked with the RhoA inhibitor confirming a role for the small GTPase in AMPH-mediated NET internalization.

**Fig. 4:**
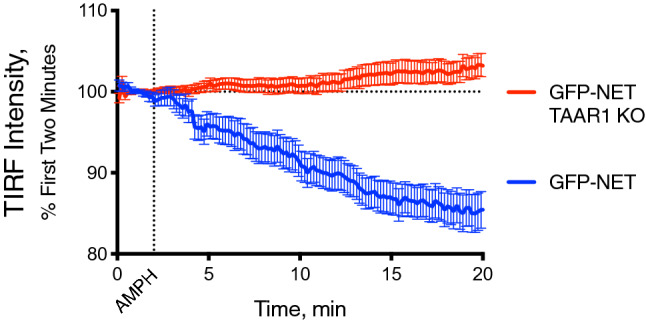
AMPH-induced NET internalization is mediated by TAAR1 receptors. In WT HEK293 cells, GFP-tagged NET internalizes in response to AMPH (10 μM) treatment (blue line). In HEK293 cells with the TAAR1 knocked out (red), AMPH has no effect on the transporter (Color figure online)

We previously demonstrated that an intracellular GPCR, TAAR1, was required for RhoA activation after AMPH treatment [3]. In that study, we generated a cell line in which the endogenous TAAR1 gene was deleted from HEK293 cells using CRISPR-Cas9 technology [3, 9]. Using this cell line along with “wildtype” HEK293 cells that have intact endogenous TAAR1 expression, we assessed AMPH-induced trafficking of a GFP-tagged NET by TIRF microscopy (Fig. 4). As seen in Fig. 1, NET was internalized after exposure to AMPH in wild-type HEK293 cells, however, in the TAAR1 KO cells there was no AMPH-dependent GFP-NET internalization. These findings demonstrate that TAAR1 is an obligate target for the effects of AMPH on NET endocytosis, similar to its role in the trafficking of DAT and EAAT3.

### CaMKII Activation is Required for AMPH-induced Catecholamine Transporter Internalization, but not for EAAT3 Internalization or RhoA Activation

**Fig. 5:**
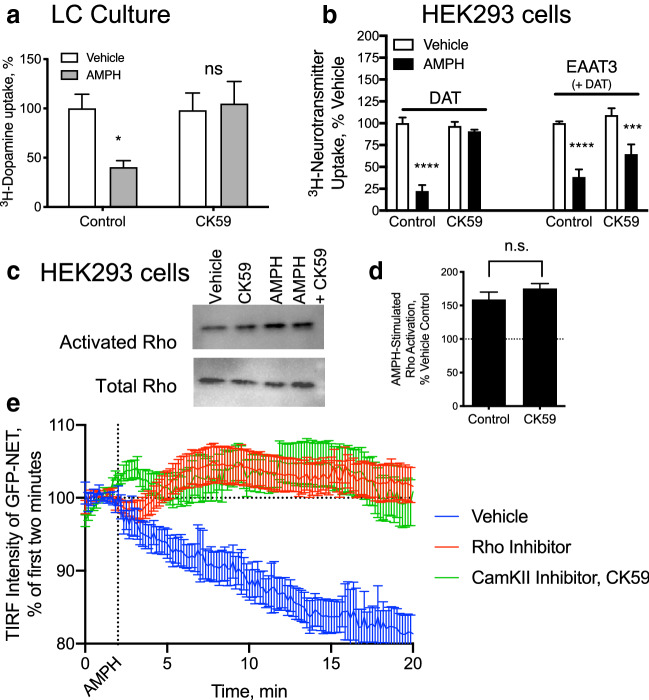
AMPH-mediated NET internalization depends on CaMKII and RhoA activation, independently. **a** Desipramine sensitive ^3^H-DA uptake in primary LC cultures decreases in response to a 30-min AMPH (10 μM) pretreatment. Co-application of the CaMKII inhibitor, CK59, (100 uM) during the pre-treatment period blocked the effects of AMPH on NET-mediated ^3^H-DA uptake. **b** HEK293 cells transiently transfected with DAT or with DAT and EAAT3 were examined for their DA or Glutamate uptake, respectively. Both transporters lost function in response to AMPH pretreatment. CK59 pretreatment, however, only significantly blocked the effects of AMPH on DAT and EAAT3 sensitivity to AMPH remained. **c**, **d** HEK293 cells expressing DAT were treated with AMPH which lead to an increase in activated RhoA, as observed previously. The CaMKII inhibitor had no effect on the Rho activation. **e** GFP-tagged NET internalization was measured by TIRF microscopy. The loss of membrane localized NET in response to AMPH was blocked by both a Rho inhibitor (red) as well as the CaMKII inhibitor, CK59 (green) (*p < 0.05, **p < 0.01, ***p < 0.001, ****p < 0.0001 by two-way ANOVA compared to vehicle control. F_(1, 30)_ = 4.436 for **a**, F_(3, 62)_ = 9.516 for **c**) (Color figure online)

Earlier studies have found that phosphorylation activities of the Ca^2+^/calmodulin-dependent protein kinase II (CaMKII) is a critical component of DAT [10, 11] and NET [6, 12] trafficking in response to AMPH in various cell models, but we wanted to address if this was true in our cultured neurons. We examined desipramine-sensitive ^3^H-DA uptake in LC cultures and found that the NET trafficking in response to AMPH could be blocked with the CaMKII inhibitor CK59, indicating a role for CaMKII in NE neurons as well (Fig. 5a).

We further investigated the role of CaMKII in AMPH-induced neurotransmitter transporter trafficking of DAT and EAAT3. EAAT3 is also internalized in response to AMPH through a RhoA-mediated [4] and TAAR1-dependent [3] mechanism in DA neurons. In HEK293 cells transfected with either DAT or DAT and EAAT3 we measured ^3^H-DA or ^3^H-Glu uptake after 30  min of AMPH pretreatment. In each case, the transport activity of the transfected carriers was decreased by AMPH pretreatment. While DAT internalization was blocked by the CaMKII inhibitor CK59, EAAT3 trafficking was not dependent upon CaMKII activation (Fig. 5b). This suggests that AMPH-mediated RhoA activation, upon which EAAT3 is also dependent, is independent of CaMKII activation.

There are indications that in other systems, CaMKII activation can directly stimulate RhoA [13], so we directly examined the potential role of CaMKII in RhoA activity in response to AMPH. We measured RhoA activation with a pull-down assay that recognizes only GTP-bound RhoA from cell lysates. Similar to our previous observations [1] we detected AMPH-induced activation in HEK293 cells expressing DAT (Fig. 5c and d). This increase in activity was unaltered by CaMKII inhibition, confirming that CaMKII activation is not upstream of RhoA activation.

AMPH-induced CaMKII activation has been implicated in the function of DAT [10] as well as internalization [11]. In order to confirm that CaMKII was altering NET-mediated trafficking rather than just transport capacity, AMPH-induced internalization of GFP-tagged NET was examined in HEK293 cells with TIRF microscopy. Inhibition of RhoA or CaMKII blocked AMPH-induced trafficking of NET.

### Calcium is Necessary for RhoA Activation

**Fig. 6:**
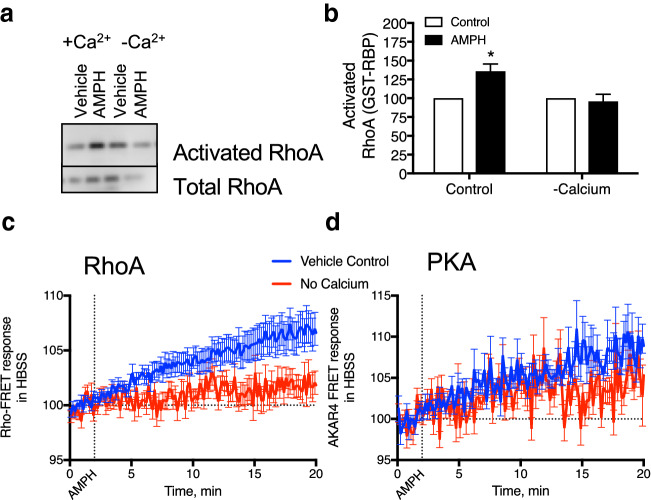
RhoA activation is dependent upon calcium **a**, **b** HEK293 cells expressing DAT were treated with AMPH (10 μM) in regular media or that without calcium and the intracellular calcium chelator BAPTA-am. RhoA activation was absent in the cells treated under calcium-free conditions. **c**, **d** Similarly, RhoA activation detected with the RhoA FRET sensor was absent in the calcium-free conditions. However, PKA activation was still observed in these cells (*p < 0.05 by two-way ANOVA compared to vehicle control. F_(1, 8)_ = 8.826) (Color figure online)

We next examined the role of calcium in AMPH-induced activation of RhoA. In HEK293 cells expressing DAT, RhoA activation could be detected in response to AMPH (Fig. 6a and b). We removed the extracellular calcium and added an intracellular calcium chelator, BAPTA-AM, and were able to block AMPH-mediated stimulation of RhoA.

We also examined the temporal response of our cells in calcium-free conditions. With a fluorescence resonance energy transfer (FRET) sensor designed to detect activated RhoA [14] we measured activation of the GTPase in response to AMPH with and without calcium (Fig. 6c). Under calcium-free conditions (red line) we did not see activation of RhoA in response to AMPH. However, in parallel assays we did find AMPH-induced activation of protein kinase A (PKA) as detected with the PKA FRET-sensor AKAR4 [15] was independent of calcium (Fig. 6d).

### EAAT3 is Internalized in LC Neurons in Response to AMPH

**Fig. 7:**
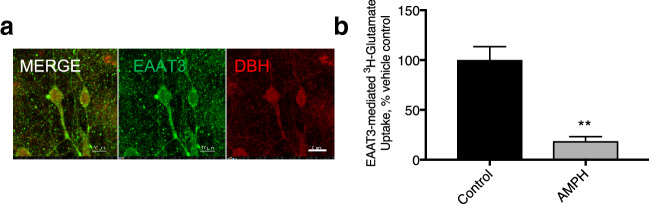
EAAT3 internalization in NE neurons Glutamate transporters in NE neurons **a** DBH(+) neurons (red) express EAAT3(+) (green). **b** EAAT3 mediated ^3^H-glutamate transport was substantially decreased in these cultures. DHK-sensitive (EAAT2-mediated) and sodium-independent glutamate uptake were unaffected by AMPH pretreatment in these cultures and have been subtracted from these data (**p < 0.01 by paired T-test) (Color figure online)

EAATs 1, 2 and 3 are the major mammalian glutamate transporters. In our LC cultures, we found that DBH (+) cells were also positive for the EAAT3 by immunolabeling (Fig. 7a). An astrocyte monolayer supports the neurons in our cultures and they were EAAT2(+), while the neurons were not. We did not detect EAAT1 immunolabeling in our cultures (data not shown).

We performed glutamate uptake assays on these cultures and found that 50.2 ± 7.34% of glutamate uptake that we measured was carried out by sodium-independent processes that could not be attributed to the EAATs. This non-specific binding/transport was not affected by AMPH pretreatment (data not shown). UCPH101 is an EAAT1 specific inhibitor that we found had no effect on glutamate uptake in the LC cultures indicating that there was no functional EAAT1 carriers, consistent with the absence of immunolabeling for the predominantly glial/cerebellar carrier in these cultures.

Dihydrokainate (DHK) is an EAAT2 specific inhibitor. We were able to block 15.6 ± 5.43% of glutamate uptake in our LC cultures with use of this inhibitor, indicating that the EAAT2 in our astrocytes in these cultures is contributing to the clearance of glutamate. EAAT2 function was unaffected by the pretreatment of these cells with AMPH (data not shown, p = 0.5420). However, the remaining sodium-dependent uptake that was not DHK sensitive can be attributed to the glutamate transporter, EAAT3. This value was dramatically decreased in response to pretreatment with AMPH indicating EAAT3 internalizes in response to AMPH in NE neurons.

## Discussion

**Fig. 8:**
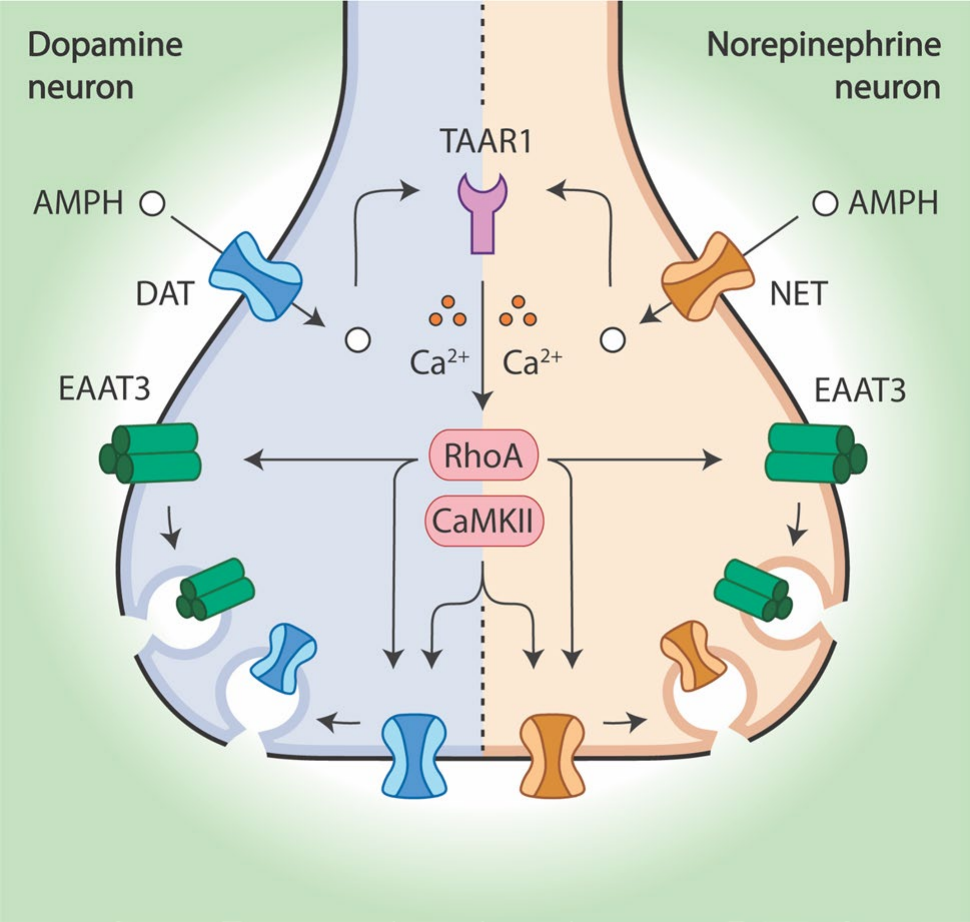
In dopamine and norepinephrine neurons, AMPH initiates a common signaling cascade that results in the endocytosis of both EAAT3 and each neuron’s respective monoamine transporter. AMPH enters dopamine neurons through DAT and norepinephrine neurons through NET. Next, AMPH activates TAAR1 which leads to the calcium-dependent activation of RhoA. This small GTPase is responsible for mediating the endocytosis of EAAT3, DAT, and NET from the plasma membrane. However, CaMKII activity is also necessary for DAT and NET internalization

We have shown here in noradrenergic neurons, cultured from the locus coeruleus (LC), that AMPH can cause trafficking of not only NET (Fig. 1), but also the neuronal glutamate transporter, EAAT3 (Fig. 7). This mechanism follows a similar sequence of events that we found for the effect of AMPH on the DAT in DA neurons. First, the AMPH is transported into the cell through the catecholamine carrier. Once inside the cell, AMPH binds to TAAR1 (Fig. 4) and triggers the activation of RhoA, which is necessary but not sufficient for the internalization of NET (Fig. 3). Like AMPH-mediated endocytosis of the DAT, NET internalization also requires activation of CaMKII in primary neurons (Fig. 5a). However, both activation of RhoA (Fig. 6) and endocytosis of EAAT3 (Fig. 7) are independent of CaMKII. RhoA activation, EAAT3/DAT/NET internalization and CaMKII activation all require calcium, indicating that mobilization of calcium is upstream of all other events and suggesting that calcium modulates RhoA activation during the intracellular signaling events activated by AMPH (Fig. 8).

### TAAR1 is an Obligate Target for the Effects of AMPH on Transporter Trafficking in NE Neurons

Previous studies have reported that TAAR1 is not highly expressed in NE neurons [16] and TAAR1 agonists were not anticipated to have a significant effect in LC cells. However, we have found that even low levels of TAAR1 expression in HEK293 cells is sufficient to initiate a robust cascade of intracellular signaling events. In TAAR1 knockout HEK293 cells, AMPH-dependent RhoA activation is absent and no internalization of DAT and EAAT3 can be detected [3]. Notably, in HEK293 cells, in LC culture and in midbrain DA neurons, the endogenous TAAR1 signals from an intracellular location and AMPH must enter the cell to activate the both PKA and RhoA signaling pathways. Thus, AMPH, DA, NE, trace amines, and other TAAR1 agonists must be present within the cell—either through endogenous synthesis, transport or membrane permeation—to regulate RhoA and PKA signaling and transporter internalization. This implies that the different behavioral effects of AMPH and AMPH-like drugs in vivo may arise from their ability to gain access to the cytoplasm through DAT, NET, SERT or other processes. We propose that the agonists’ entry into the cell and accessibility to the intracellular TAAR1 may be an explanation for some of the selective actions of different psychostimulants. Our demonstration that signaling by intracellular TAAR1 contributes to the actions of AMPH in LC noradrenergic neurons, as well as midbrain dopamine neurons, provides additional evidence that this mechanism may operate broadly to regulate cellular responses in other neuronal populations.

### AMPH Can Regulate Excitatory as Well as Catecholaminergic Signaling in LC Neurons

We previously showed that regulation of the trafficking of a glutamate EAAT3 plays a critical role in regulating glutamatergic inputs into these cells; internalization of the glutamate transporter in response to AMPH leads to enhanced glutamatergic responses in DA neurons [4]. Thus, removal of EAAT3 from the surface of NE neurons could potentiate glutamatergic inputs onto NE neurons. Projections to the LC are from all over the brain, several of which are glutamatergic [17, 18] that may also be potentiated by this modulation in NE neurons. Further exploration of these specific inputs may be important.

### Implications for Signaling by NE Neurons

In light of increasing reports of release of multiple neurotransmitters from the same neuron [19], it is possible that LC neurons are capable of packaging glutamate into vesicles for subsequent release. Glutamate co-release from LC neurons has been reported [20] and internalization of EAAT3 by AMPH could limit local glutamate entry and alter capacity for release. The glutamate transported into these cells by EAAT3 may also modulate GABA-co-transmission. The glutamate that EAAT3 transports into GABA-ergic cells serves as an important precursor for GABA synthesis; blocking the transporter decreases inhibitory GABA-ergic neurotransmission [21]. For LC neurons, GABA co-release could also be modulated through EAAT3 trafficking.

In addition, EAAT3 is important for the oxidative balance of neurons since it also transports cysteine, a rate limiting component of glutathione, an important component of baseline oxidative balance in neurons [22]. DA neurons are particularly sensitive to the loss of EAAT3 expression and knockout animals exhibit substantial oxidative cell-death [23]. Loss of glutathione in NE neurons may disrupt the overall oxidative balance and cellular health, and a reduction in surface EAAT3 following AMPH administration could make cells more vulnerable to other perturbations.

Finally, there is also potential for additional crosstalk with the dopamine system. Most of the NE cell bodies originate in the LC and project to the hippocampus or cortex, although there are several other targets as well. At these targets, it is unlikely that NET clears only NE. NE neurons release significant amounts of DA along with NE, and the NET has been shown to effectively transport DA in the hippocampus [24] frontal cortex and to a lesser extent in the caudate and nucleus accumbens [25]. AMPH and other TAAR1 agonists that activate the internalization of NET in these sites may contribute to alterations DA signaling in response to these drugs. Overall, the observation in NE neurons that AMPH activates signaling pathways through an intracellular GPCR, TAAR1, to regulate NET and EAAT3 trafficking provides new avenues and targets for selectively modulating noradrenergic neurotransmission. However, much work remains to resolve the precise signaling pathways, where they occur within NE neurons and ultimately, how TAAR1 regulates NE neuron physiology and function.

## Materials and Methods

### Cell Lines

HEK293 cells were obtained from ATTC and maintained in DMEM with 5% fetal bovine serum and Penn/Strep antibiotics and maintained in 5% CO_2_. TAAR1 KO HEK293 cells were derived with CRISPR-Cas9 as described previously [3]. Cells were transfected with Lipofectamine 2000 according to the manufacturer’s instructions (Invitrogen) and plated on poly-l-lysine coated coverslips or plates. For calcium-free assays, cells were plated on Matrigel (BD Biosciences).

### Primary Culture

E15 Swiss Webster tissue from the VTA or the LC was isolated, gently triturated and plated at a density of 3 tissue samples per 12, 12 mm coverslips or eight MatTek dishes. Glass surfaces were coated with poly-d-lysine. Cultures were maintained for 2–5 weeks at 5% CO_2_ in 5% FBS, 5% FCS in MEM.

All assays were done in accordance with the National Institutes of Health Animal Care Committee.

### ^3^H-neurotransmitter Uptake Assays

DAT, NET and EAAT3 function were assessed with ^3^H-neurotransmitter uptake assays. 20 μM cold and 100 nM ^3^H-dopamine or 250 μM cold and 50 nM ^3^H-glutamate were applied to cells for 10 min in PBS supplemented with Mg^2+^ and Ca^2+^. Non-transported, extracellular neurotransmitter was washed off with PBS. Cells were lysed in scintillation fluid and cpms were determined on a β-counter. In this, as with all cell culture assays, the procedure was performed from transfection to uptake at least three independent times. A single well is considered n for statistical purposes.

### Biotinylation Assays

HEK293 cells transiently transfected with GFP-DAT or GFP-NET were washed with ice-cold PBS twice and chilled on ice for 10 min. Then cells were incubated with 3.30 mM sulfo-NHS-SS-biotin (Pierce) in biotinylation buffer (in mM: 2 Cacl2, 150 NaCl, and 10 triethanolamine, pH 7.5) at 4 °C for 40 min. Next, cells were washed twice with ice-cold PBS and the reaction was quenched with 100 mM glycine in PBS at 4 °C for 20 min. Cells were washed once with ice-cold PBS and incubated in lysis buffer (1% Triton-X, 150 mM NaCl, 5 mM EDTA, 50 mM Tris) at 4 °C for 20 min. Lysates were centrifuged at 13,000 rpm at 4 °C for 10 min, protein concentrations were normalized with BCA protein assay (Thermo Fisher) and analyzed by western blot probed with an antibody to GFP.

### Immunochemistry

Cells were fixed with 4% paraformaldehyde for 20 min at 4 °C, permeabilized with 0.125% Triton X-100 in PBS and blocked with True Black (Biotium) according to the manufacturer’s directions and then 10% normal goat serum (NGS) for 1 h. Primary antibodies were applied overnight in 5% NGS at 1:1000. Secondary antibodies were applied for 1–2 h at room temperature, diluted 1:1000 in 5% NGS. Coverslips were then mounted in Fluoromount Aqueous Mounting Medium (Sigma). Antibodies used in this study include rabbit anti-GFP (Clontech, 612460), rat anti-DAT (Millipore MAB369), sheep anti-DBH (abcam ab19353), rabbit anti-RhoA (abcam, ab54835), rabbit anit-NeuN (Abcam, ab177487) and rabbit anti-EAAT3 (α-Diagnostics, EAAC11-A).

### GST Pulldown Assays

GST conjugated to the RhoA binding domain of Rhotekin (GST-RBD) were purified from *E. coli* and bound to glutathione-Sepharose beads [26]. Transiently transfected HEK293 cells were treated with experimental conditions and lysates were prepared in binding buffer (50 mM Tris Cl, pH 7.2, 1% Triton X-100, 500 mM NaCl, 10 mM MgCl_2_). Activated Rho proteins were captured overnight at 4°C and subsequently analyzed by western blot.

### Microscopy

Confocal microscopy, FRET and TIRF imaging was performed on a Nikon A1Rsi confocal/TIRF microscope. For all imaging assays, cells were transfected and imaged 12–48 h later. For FRET assays, CFP and YFP images were collected under 405 nm illumination. For time-lapse assays, after background subtraction, data were normalized to the 2 min prior to treatment. Selection criteria for TIRF images was fluorescent stability (less than 10% variation) for at least 2 min before drug application. For RhoA and PKA/AKAR4 FRET sensor assays, selection criteria included a positive response to Rho Activator I (Cytoskeleton) or epinephrine at the end of the experiment.

### Quantitation and Statistics

Western blots were quantified by densitometry with FIJI. Statistical analysis was carried out with Graphpad Prism 6 as described in the figure legends. Where possible, experimenters were blinded to the conditions being tested. All values in the figures indicate mean ± SEM.
